# Tumor Volume Predicts High-Risk Patients and Guides Initial Chemoradiotherapy for Early Cervical Cancer

**DOI:** 10.3389/fonc.2021.640846

**Published:** 2021-04-27

**Authors:** Jingjing Zhang, Dongyan Cao, Jiaxin Yang, Keng Shen, Yonglan He, Huadan Xue

**Affiliations:** ^1^ Department of Gynecology and Obstetrics, The First Affiliated Hospital, Sun Yat-sen University, Guangzhou, China; ^2^ Department of Obstetrics and Gynecology, Peking Union Medical College Hospital, Chinese Academy of Medical Science and Peking Union Medical College, Beijing, China; ^3^ Department of Radiology, Peking Union Medical College Hospital, Chinese Academy of Medical Science and Peking Union Medical College, Beijing, China

**Keywords:** cervical cancer, tumor-free margin, tumor volume, adverse pathological risk factor, predictive value, high-risk patients

## Abstract

We evaluated the relationship between the minimum tumor-free margin, tumor volume, and adverse pathological risk factors in early cervical cancer and explored the predictive value of these parameters for different types of risk patients to guide individualized therapeutic strategies. Patients who received the initial treatment of radical operation of cervical cancer and their postoperative pathological reports in our hospital from July 1, 2017, to June 30, 2019, were reviewed. Their minimum tumor-free margin and tumor volume were measured on preoperative magnetic resonance imaging. Student’s t-test and the receiver operating characteristic curve analysis were used for data analysis. A total of 240 patients were included. Adverse pathological risk factors were as follows: deep cervical infiltration, 95 (39.6%) cases; lymph vascular space invasion, 91 (37.9%); lymph node metastasis, 20 (8.3%); parametrial infiltration, 8 (3.3%); tumor diameter ≥4 cm, 7 (2.9%); and positive surgical margin, 1 (0.4%). According to the adverse pathological factors, there were 20 (8.3%) high-risk patients, 50 (20.8%) medium-risk patients, and 170 (70.8%) low-risk patients. The ranges of the minimum tumor-free margin and tumor volume were 0.01–13.5 mm and 105–27,990 mm^3^, respectively. The minimum tumor-free margin with lymph node metastasis was significantly smaller than that without (P <0.05). The tumor volume with parametrial infiltration, deep cervical infiltration, or lymph vascular space invasion was significantly greater than that without (P < 0.05). The tumor volume was significantly different among low-, medium-, and high-risk patients (P <0.05). Tumor volume was of predictive value for high-risk patients (P < 0.05). With 3,505 mm^3^ as the cutoff value, the sensitivity and specificity for the prediction of high-risk patients were 88.9% and 84.8%, respectively. Tumor volume can be used as a great predictor of high-risk patients (cutoff value, 3,505 mm^3^), which could be an indication of initial chemoradiotherapy for early cervical cancer.

## Introduction

As the fourth most common cancer among women worldwide, cervical cancer represents an important female health challenge (1). The International Federation of Gynecology and Obstetrics (FIGO) for cervical cancer is a clinical staging system; gynecological examination can better evaluate parametrial infiltration and vaginal involvement, whereas the use of the clinical staging system can prevent advanced cervical cancer patients from undergoing surgery (2).

Tumor size has always been an important indicator of cervical cancer staging since 1928 when cervical cancer staging literature can be traced (3). Tumor diameter is most widely used to represent the tumor size of cervical cancer. In stage IB of the FIGO staging system (2018), a tumor diameter ≥2 cm was incorporated as a new cutoff value of stages IB1 and IB2, and a tumor diameter ≥4 cm was characterized as stage IB3 (4). However, a complex irregular three-dimensional (3D) configuration, but not a regular ellipsoid shape, of cervical cancer and the variant location of the tumor on the cervix can make an accurate evaluation of tumor diameter difficult. An alternative parameter, the minimum tumor-free margin, is a measure of the minimum distance between the edge of the tumor and cervix. Additionally, tumor volume has been suggested as a potential parameter for assessing tumor size and prognosis (5).

This study aimed to explore the relationship between the minimum tumor-free margin, tumor volume, and adverse pathological risk factors in early cervical cancer and the predictive value of these parameters for medium- or high-risk cervical cancer patients who were at risk of adjuvant chemoradiotherapy, which can be very useful to make the decision of initial chemoradiotherapy and avoid unnecessary surgery and potential complications.

## Materials and Methods

### Ethics Statement

Ethics approval was granted by the institutional review board (approval number: S-K910). The informed consent for study participation was waived.

### Study Design and Population

**Figure 1:**
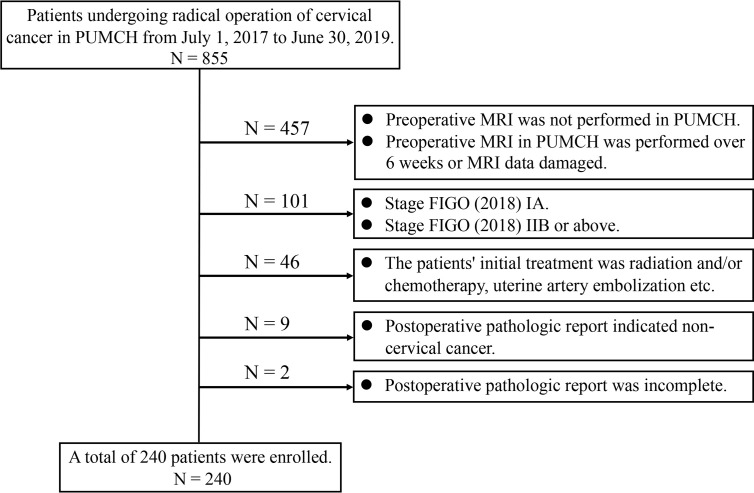
Flow chart of enrolled patients. PUMCH, Peking Union Medical College Hospital; MRI, magnetic resonance imaging; FIGO, International Federation of Gynecology and Obstetrics.

A total of 855 patients were retrieved from the medical records department of our hospital under the condition of “Patients undergoing the radical, subradical, or extrafascial hysterectomy or radical trachelectomy plus lymphadenectomy at Peking Union Medical College Hospital from July 1, 2017, to June 30, 2019.” Patients were enrolled if they met the following inclusion criteria: after the stage at diagnosis was corrected according to the FIGO (2018), stage IB to IIA was identified; the patient underwent magnetic resonance imaging (MRI) at our hospital within 6 weeks preoperatively; the patient’s initial treatment was one of the aforementioned surgeries in our hospital; and the patient had complete pathological data. Patients were excluded if they met the following exclusion criteria: FIGO (2018) stages IA and IIB or above; preoperative MRI was not performed at our hospital; preoperative MRI at our hospital was performed over 6 weeks, or MRI data were damaged; the patient’s initial treatment was radiation and/or chemotherapy, uterine artery embolization, etc.; and the postoperative pathological report indicated non-cervical cancer or it was incomplete. A total of 240 patients were enrolled according to the inclusion and exclusion criteria (**Figure 1**).

### Data Collection and Analysis

The clinical-pathological characteristics of enrolled patients, including age, FIGO stage, type of operation, pathological results, and postoperative adjuvant therapy, were collected.

According to the pathological report of each patient, six adverse pathological risk factors, including (1) lymph node metastasis (2), parametrial infiltration (3), positive surgical margin (4), deep cervical infiltration (infiltration depth >1/2 of the cervical wall thickness) (5), lymph vascular space invasion, and (6) lesion diameter ≥ 4 cm were extracted and analyzed. If any one of (1) to (3) was positive, the patient was defined as being high-risk; if any two of (4) to (6) were positive, the patient was defined as medium-risk; if the above conditions were not met, the patient was defined as being low-risk.

The pelvic T1-weighted imaging sequence and high-resolution T2-weighted imaging sequence were scanned by a 1.5T magnetic resonance scanner (General Electric Company) with a slice thickness of 5 mm and 1.5 mm separation. The contrast agent was intravenously injected into the patient with gadolinium-dextran injection (20 mL: 9.38 g, Beijing Beilu Pharmaceutical Co., Ltd.) at a dose of 0.1 mmol/kg.

A radiologist with approximately 10 years of experience reviewing MRI measured the two parameters. In transverse, coronal, and sagittal planes of high-resolution T2-weighted MRI, the minimum vertical width between the cervical lesion and cervical lateral margin was measured and defined as the minimum tumor-free margin of this plane, and the minimum value among tumor-free margin values measured on three planes was taken as the minimum tumor-free margin of the lesion. This process was performed twice. In each sagittal plane of the high-resolution T2-weighted MR image, the edge of the cervical cancer lesion was outlined, and the data were uploaded to InferScholar^®^ Center of Infervision, an artificial intelligence research platform. The platform automatically calculates the volume of the cervical cancer lesion.

SPSS software version 25.0 (IBM Corp.) was used for statistical analysis. P <0.05 was considered statistically significant. Two groups of measurements were compared using Student’s t-test; multiple groups of measurements were assessed by one-way ANOVA. The predictive value of measurements and the optimal cutoff threshold were determined by calculating the area under the receiver operating characteristic (ROC) curve.

## Results

Patients’ clinical-pathological characteristics are shown in **Table 1**. The average age of enrolled patients was 43.8 (range, 24–80) years. The common pathological types of cervical cancer, including squamous cell carcinoma, adenocarcinoma, and adenosquamous carcinoma, accounted for 94.2% of the enrolled patients. Other pathological types included cervical neuroendocrine carcinoma (three patients), cervical small cell carcinoma (two patients), undifferentiated cervical carcinoma (two patients), clear cell carcinoma (two patients) and malignant melanoma (two patients), cervical sarcoma (one patient), lymphoepitheliomatoid carcinoma (one patient), and mixed carcinoma (small cell neuroendocrine carcinoma and squamous cell carcinoma in situ) (one patient). All patients underwent radical, subradical, or extrafascial hysterectomy or radical trachelectomy plus lymphadenectomy; there were 208 (208/240, 86.7%) patients who underwent operation through the laparoscopic approach and 32 (32/240, 13.3%) through the open approach; 209 (209/240, 87.1%) patients underwent pelvic lymphadenectomy, and 31 (31/240, 12.9%) underwent para-aortic and pelvic lymphadenectomy. A total of 114 (114/240, 47.5%) patients received postoperative adjuvant therapy, including nine (9/240, 3.75%) who received only adjuvant chemotherapy, 14 (14/240, 5.8%) who received only adjuvant radiotherapy, and 92 (92/240, 38.3%) who received adjuvant chemoradiotherapy. In addition to 70 (70/114, 61.4%) medium- or high-risk patients, 44 (44/240, 38.6%) low-risk patients received postoperative adjuvant therapy for their rare pathological types or positivity of deep cervical infiltration, lymph vascular space invasion, or lesion diameter ≥ 4 cm.

**Table 1:** Patients’ clinical-pathological characteristics (N = 240).

Clinical-pathological characteristics	n (%)
Age [years; M ± SD (range)]	43.8 ± 10.2 (24-80)
Histology	Squamous: 166 (69.2%) Adeno- and adenosquamous: 60 (25%) Other: 14 (5.8%)
FIGO stage (2018)	IB1: 128 (53.3%) IB2: 80 (33.3%) IB3: 16 (6.7%) IIA1: 10 (4.2%) IIA2: 6 (2.5%)
Operation approach	Laparoscopic approach: 208 (86.7%) Open approach: 32 (13.3%)
Type of hysterectomy	Radical hysterectomy: 219 (91.3%) Subradical hysterectomy: 8 (3.3%) Extrafascial hysterectomy: 2 (0.8%) Radical trachelectomy: 11 (4.6%)
Type of lymphadenectomy	Pelvic lymphadenectomy: 209 (87.1%) Paraaortic and pelvic lymphadenectomy: 31 (12.9%)
Postoperative adjuvant therapy	No: 126 (52.5%) Only chemotherapy: 9 (3.75%) Only RT: 14 (5.8%) Chemoradiotherapy: 92 (38.3%)
Adverse pathological risk factors	(1) Lymph node metastasis: 20 (8.3%) (2) Parametrial infiltration: 8 (3.3%) (3) Positive surgical margin: 1 (0.4%) (4) Deep cervical infiltration: 95 (39.6%) (5) Lymph vascular space invasion: 91 (37.9%) (6) Lesion diameter ≥4 cm: 7 (2.9%)
Risk grading	Low-risk patients: 170 (70.8%) Medium-risk patients: 50 (20.8%) High-risk patients: 20 (8.3%)

M, mean; SD, standard deviation; FIGO, International Federation of Gynecology and Obstetrics; RT, radiotherapy.

The minimum tumor-free margin was measured in 66 (66/240, 27.5%) patients. The minimum tumor-free margin of the remaining patients cannot be measured because 58 (58/240, 24.2%) patients’ cervical cancer lesions reached the external cervical os, 36 (36/240, 15.0%) patients’ cervical cancer lesions involved the vaginal fornix, and 80 (80/240, 33.3%) patients’ MR image did not show cervical cancer lesions because some stage IB1 lesions, as small as few millimeters, failed to show on MRI. The intra-class correlation coefficient of the same radiologist for the repeatability evaluation of the minimum tumor-free margin was 0.995 (95% confidence interval [CI], 0.992–0.997). The mean minimum tumor-free margin ranged from 0.01 to 13.5 mm, with a median value of 2.1 mm (Q1, 1.3 mm; Q3, 4.7 mm). A total of 160 (160/240, 66.7%) patients’ MR image showed cervical cancer lesions, and 134 (134/160, 83.8%) patients’ high-resolution T2-weighted MR image outlined the edge of the cervical cancer lesion. The tumor volume was measured in 134 (134/240, 55.8%) patients, and it ranged from 105 to 27,990 mm^3^, with a median value of 1,626 mm^3^ (Q1, 904 mm^3^; Q3, 4,651 mm^3^). The minimum tumor-free margin and volume of lesions at different stages were statistically different (P <0.01 and <0.001, respectively).

**Figure 2:**
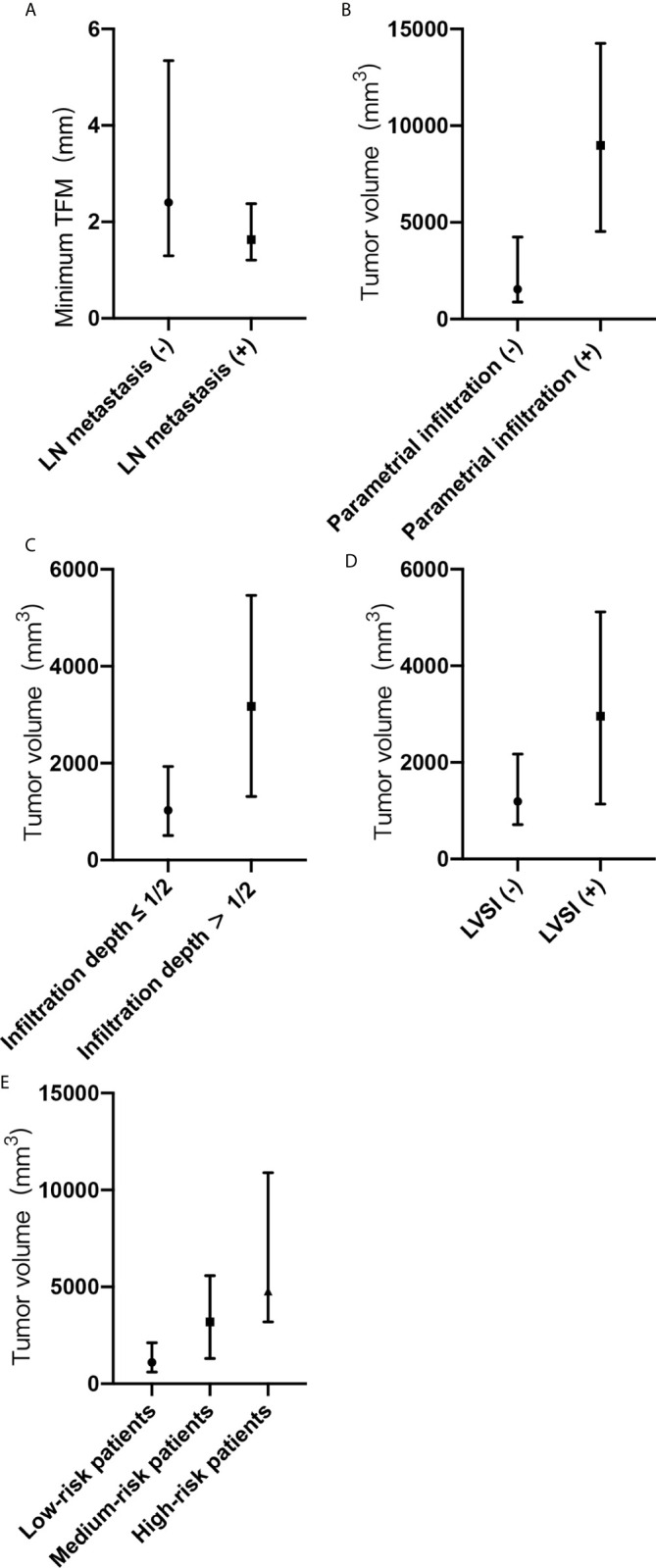
**(A-E)** Minimum tumor-free margin (TFM) and tumor volume with adverse pathological risk factors and risk grading. **(A)** Minimum TFM in groups with or without lymph node metastasis. **(B)** Tumor volume in groups with or without parametrial infiltration. **(C)** Tumor volume in groups with or without deep cervical infiltration. **(D)** Tumor volume in groups with or without lymph vascular space invasion. **(E)** Tumor volume in groups of low-risk, medium-risk, and high-risk patients. TFM, tumor-free margin; LN, lymph node; LVSI, lymph vascular space invasion; (-), negative; (+), positive.

Between the groups with and without lymph node metastasis, there was a statistical difference in the minimum tumor-free margin (P <0.05). The median minimum tumor-free margins were 2.4 mm (Q1, 1.3 mm; Q3, 5.3 mm) in the group without lymph node metastasis and 1.6 mm (Q1, 1.2 mm; Q3, 2.4 mm) in the group with lymph node metastasis (**Figure 2A**). Tumor volume was larger in the group with lymph node metastasis than in the group without lymph node metastasis (3,983 mm^3^ [Q1, 1,514 mm^3^; Q3, 4,861 mm^3^] vs. 1,538 mm^3^ [Q1, 885 mm^3^; Q3, 4,278 mm^3^]). However, there was no statistical difference in the tumor volume between the groups (P > 0 .05).

Between the groups with and without parametrial infiltration, there was a statistical difference in the tumor volume (P <0.05). The median tumor volumes were 1,545 mm^3^ (Q1, 885 mm^3^; Q3, 4,247 mm^3^) in the group without parametrial infiltration and 8,979 mm^3^ (Q1, 4,527 mm^3^; Q3, 14,258 mm^3^) in the group with parametrial infiltration (**Figure 2B**). Between the groups with and without deep cervical infiltration, there was a statistical difference in the tumor volume (P <0.05). The median tumor volumes were 1,025 mm^3^ (Q1, 508 mm^3^; Q3, 1,934 mm^3^) in the group of cervical infiltration depth ≤1/2 and 3,175 mm^3^ (Q1, 1,311 mm^3^; Q3, 5,462 mm^3^) in the group of cervical infiltration depth >1/2 (**Figure 2C**). Between the groups with and without lymph vascular space invasion, there was a statistical difference in the tumor volume (P <0.05). The median tumor volumes were 1,282 mm^3^ (Q1, 714 mm^3^; Q3, 2,258 mm^3^) in the group without lymph vascular space invasion and 2,958 mm^3^ (Q1, 1,137 mm^3^; Q3, 5,116 mm^3^) in the group with lymph vascular space invasion (**Figure 2D**). The median tumor volumes showed significant differences according to risk grading (P <0.05) and were as follows: low-risk patients, 1,118 mm^3^ (Q1, 589 mm^3^; Q3, 2,128 mm^3^); medium-risk patients, 3,188 mm^3^ (Q1, 1,306 mm^3^; Q3, 5,581 mm^3^); and high-risk patients, 4,776 mm^3^ (Q1, 3,192 mm^3^; Q3, 10,889 mm^3^) (**Figure 2E**).

**Figure 3:**
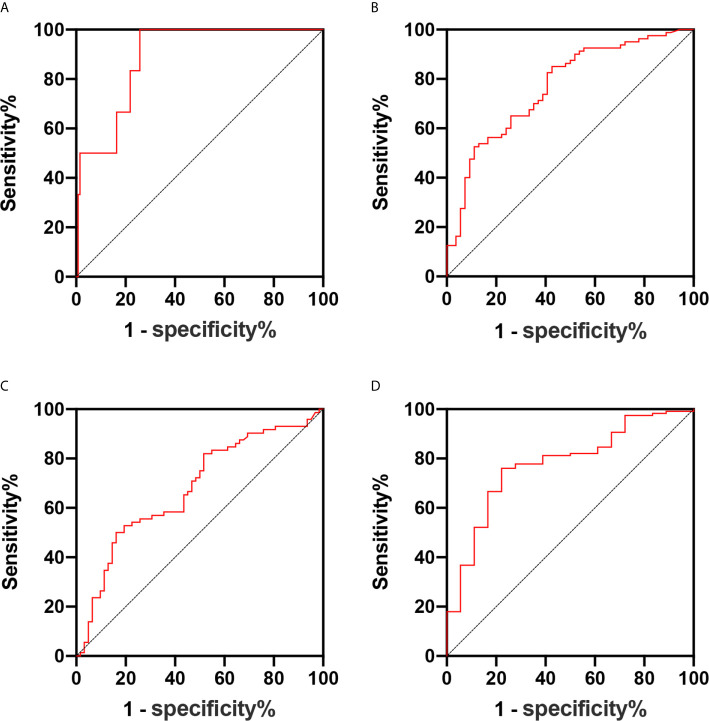
**(A–D)** Receiver operating characteristic curve of the predictive values of the tumor volume for parametrial infiltration **(A)**, deep cervical infiltration **(B)**, lymph vascular space invasion **(C)**, and high-risk patients **(D)**.

The tumor volume was associated with the presence of parametrial infiltration, deep cervical infiltration, and lymph vascular space invasion (all, P <0.05). For parametrial infiltration prediction, the cutoff value for the tumor volume was 3,882 mm^3^, with a sensitivity of 100% and specificity of 74.2% (area under the ROC curve, 0.888; 95% CI, 0.795–0.981) (**Figure 3A**). For deep cervical infiltration prediction, the cutoff value was 2,881 mm^3^, with a sensitivity of 52.5% and specificity of 88.9% (area under the ROC curve, 0.766; 95% CI, 0.684–0.847) (**Figure 3B**). For lymph vascular space invasion prediction, the cutoff value was 1,072 mm^3^, with a sensitivity of 81.9% and specificity of 46.8% (area under the ROC curve, 0.667; 95% CI, 0.575–0.760) (**Figure 3C**). According to the risk classification of patients with six adverse pathological risk factors, the tumor volume was the predictive value of high-risk patients (P <0.05). For high-risk patient prediction, the cutoff value was 3,505 mm^3^, with a sensitivity of 88.9% and specificity of 84.8% (area under the ROC curve, 0.841; 95% CI, 0.68–1) (**Figure 3D**).

## Discussion

Surgery is the main treatment for early cervical cancer; cervical conization, simple hysterectomy, radical or subradical hysterectomy plus lymphadenectomy, or radical trachelectomy plus lymphadenectomy may be selected according to the disease stage and patients’ request to preserve fertility. Based on postoperative adverse pathological risk factors, adjuvant radiotherapy and/or chemotherapy may be added postoperatively (6, 7).

Cervical cancer stages IB and IIA can be cured equally effectively with radical surgery or chemoradiotherapy. However, the two procedures differ in associated morbidity and type of complications. Combining surgery with chemoradiotherapy leads to a serious issue that patients experience not only surgical complications but also have various short- and long-term chemoradiotherapy toxicities (8, 9). Recently, the ABandoning RAd hyst in cerviX cancer (ABRAX) trial, the biggest retrospective analysis of 515 patients to date, conducted by Cibula et al. (10), showed that there is no difference in the oncologic outcome of patients where a lymph node involvement was diagnosed intraoperatively if the hysterectomy was completed or abandoned before initial chemoradiotherapy. This data supports the importance of avoiding hysterectomy when there is a clear indication for initial chemoradiotherapy.

Reliable preoperative prediction of adverse pathological risk factors to identify high- or medium-risk cervical cancer patients before initial treatment decision-making is greatly needed, which can help determine whether a patient needs initial chemoradiotherapy rather than surgery. Clinicians select the appropriate treatment for individual patients to avoid unnecessary surgical complications, reduce treatment costs, and improve patient quality of life.

However, not all cervical cancer lesions are visible to the naked eye or MRI based on the definition of cervical cancer stage IB1 (4). Moreover, the morphological variant of cervical cancer lesions greatly influenced the measurement of this parameter. Therefore, a high rate of missing measurements of the minimum tumor-free margin in our study. The finding that the minimum tumor-free margin was related to only lymph node metastasis revealed the weak relationship between the minimum tumor-free margin and adverse pathological factors. The disability in predicting lymph node metastasis further limited its usage in early cervical cancer.

In 2001-2002, two studies related to cervical cancer tumor volume were reported. A study of 30 patients conducted by Chen et al. (11) showed that the tumor volume of cervical cancer was associated with lymph node metastasis and parametrial infiltration. Another study by Wagenaar et al. (5) on 126 patients, a larger sample size, found that the parametrial infiltration was tumor volume-dependent, but lymph node metastasis was not. Both studies evaluated tumor volume based on MRI. The former study evaluated the tumor volume using two methods: the first method, tumor volume estimates, used three axial measurements or the longest axial measurement, which was equivalent to calculating a cylinder volume; the second one, 3D tumor volumetry, used the integration of tumor areas in different images from volumetric software. Compared with tumor volume estimates, 3D tumor volumetry was superior in differentiating lymph node metastasis from parametrial infiltration. The latter evaluated tumor volume by multiplying the sum of the tumor areas by the slice thickness. Among the three tumor volume measurement methods, 3D tumor volumetry and the tumor volume formula were more accurate than tumor volume estimates. Recently, a new related study was published by Chen et al. (12) where the tumor volume was assessed to predict lymph node involvement and the presence of lymphovascular space invasion in 315 patients. In this study, the method of tumor volume measurement was the same as that of Wagenaar et al. (5). The emerging 3D transvaginal ultrasonography provides an approach for the assessment of tumor volume as a low-cost alternative (13).

The tumor volume measurement method of our study is similar to 3D tumor volumetry. Although simple and convenient, the cylinder volume as a rough estimate of cervical cancer tumor volume has been abandoned. However, more accurate measurement methods of cervical cancer tumor volume based on MRI are labor-intensive and time-consuming, which require outlining the tumor area, and may need the help of other software to integrate tumor areas from different slices. Intelligent techniques and software are required to solve the dilemma.

In our study, the tumor volume was associated with parametrial infiltration, deep cervical infiltration, and lymph vascular space invasion. The parameter was larger in patients with lymph node metastasis than in those without lymph node metastasis, although the result did not reach statistical significance. The comparison of correlation between the tumor volume and adverse pathological risk factors of the aforementioned studies and our study is shown in **Table 2**. Chen et al. (12) did not mention any association between the tumor volume and parametrial infiltration, and the findings of Wagenaar et al. (5) and Chen et al. (11) are consistent with our study. In addition, our study showed that the tumor volume had a good predictive value for parametrial infiltration with a sensitivity of 100% and specificity of 74.2% (area under the ROC curve, 0.888; 3,882 mm^3^). This result has good clinical application value, that is, the initial chemoradiotherapy should be recommended for cervical cancer patients at high risk of parametrial infiltration evaluated by the tumor volume ≥ 3,882 mm^3^.

**Table 2:** The comparison of correlation between the tumor volume and adverse pathological risk factors of the above-mentioned studies and our study.

Year	Authors	Number of patients	Adverse pathological risk factors related tumor volume
2001	Wagenaar et al (5)	126	Parametrial infiltration; Deep cervical infiltration^1^
2002	Chen, A.C. et al (12)	30	Parametrial infiltration; Lymph node metastasis
2018	Chen, X.L. et al (13)	315	Lymph node metastasis; Lymph vascular space invasion
2019	Zhang J.J. et al (2)	134	Parametrial infiltration; Deep cervical infiltration; Lymph vascular space invasion; Lymph node metastasis^3^

^1^In Wagenaar et al's study, deep cervical infiltration was identified as invasion depth □ > 10 mm.

^2^The authors of our present study.

^3^In our study, the tumor volume was larger in patients with lymph node metastasis than in those without lymph node metastasis, although the result did not reach statistical significance.

The relevance of the tumor volume and lymph node metastasis is recognized generally except by Wagenaar et al. (5). The opposite result may be due to the subgroup setting of small and large tumors using a cutoff value of 35 mL for tumor volume. In our study, although the results did not reach statistical significance, the tumor volume was larger in patients with lymph node metastasis. Increasing the number of enrolled patients may result in a statistically significant difference.

Similar to Chen et al. (12), our study found that tumor volume has a predictive value in the presence of lymph vascular space invasion. The cutoff value, area under the ROC curve, and sensitivity and specificity of lymph vascular space invasion prediction in our study and in that by Chen et al. were 1,072 mm^3^, 0.667, and 81.9% and 46.8% and 6,410 mm^3^, 0.806, and 60.2% and 93.4%, respectively. The large gap between the two cutoff values and the low specificity in our study and low sensitivity in the study by Chen et al. require more data for validation.

The correlation between the tumor volume and deep cervical infiltration was only mentioned by Wagenaar et al. (5). The definition of deep interstitial invasion of cervical cancer has not been completely unified in different societies or regions (14). The most common cutoff value of deep cervical infiltration is the 1/2 or 1/3 infiltration depth, which is widely used by many studies (6, 15, 16); additionally, Covens et al. (17) chose the invasion depth of 10 mm as the criteria and Meirovitz et al. (18) 8mm. Wagenaar et al. identified deep cervical infiltration with an invasion depth >10 mm. Our study identified it as infiltration depth >1/2 of the cervical wall thickness. Based on this, tumor volume has a certain value in predicting deep cervical infiltration; however, the sensitivity, approximately 50%, limits its value in clinical application.

With careful consideration of the aforementioned adverse pathological risk factors related to tumor volume, our study showed that the tumor volume had a great predictive value for high-risk patients with a cutoff tumor volume of 3,505 mm^3^. This result reminds us that cervical cancer patients with a tumor volume ≥3.5 cm^3^ who underwent surgery as the initial treatment had a high possibility of receiving adjuvant chemoradiotherapy postoperatively. The clinical application of a tumor volume ≥3.5 cm^3^ in predicting patients’ optimal treatment is considerable. Initial chemoradiotherapy as these patients’ optimal treatment can achieve the same therapeutic effect (8), avoid the complications related to surgery, reduce the cost of treatment, and improve patients’ quality of life after treatment.

This study has several limitations. The first limitation of this study is the retrospective design. Consequently, our findings need to be verified by subsequent prospective studies involving larger populations before they are applied safely in clinical practice. Moreover, a study reported the tumor volume of cervical cancer can be applied in the outcome prediction (19). The recurrence and prognosis of our study population will be followed up to determine their relationship to tumor volume in early cervical cancer. The pelvic MRI slice thickness was 5 mm, and separation was 1.5 mm, which may have led to the partial loss of morphological information of cervical cancer lesions and reduced the accuracy of the minimum tumor-free margin and tumor volume measurements.

In conclusion, more adverse pathological factors were associated with the tumor volume than the minimum tumor-free margin. Additionally, the tumor volume has great value in predicting high-risk patients (cutoff value, 3,505 mm^3^). The measurement of tumor volume may change the methods involved in daily practice in the treatment of early-stage cervical cancer patients.

## Data Availability Statement

The original contributions presented in the study are included in the article/supplementary material. Further inquiries can be directed to the corresponding author.

## Ethics Statement 

The studies involving human participants were reviewed and approved by the Institutional Review Board (IRB) of Peking Union Medical College Hospital (PUMCH) (approval number: S-K910). Written informed consent for participation was not required for this study in accordance with the national legislation and the institutional requirements.

## Author Contributions

KS, HX, DC, and JZ developed the idea and designed the study. KS, JY, and DC were involved in the diagnosis and treatment of the enrolled patients. HX and YH measured the two parameters. JZ collected and analyzed the data. JZ and YH drafted the manuscript. All authors contributed to the article and approved the submitted version.

## Funding

This study was supported by the Fundamental Research Funds of Central Public Welfare Scientific Institution of Chinese Academy of Medical Science (2019PT320011).

## Conflict of Interest

The authors declare that the research was conducted in the absence of any commercial or financial relationships that could be construed as a potential conflict of interest.
